# Current News

**Published:** 1895-04

**Authors:** 


### Current News.

### TRI-STATE MEETING.

THE joint meeting of the Dental Societies of Ohio, Michigan, and Indiana will occur June 18, 19, 20, 1895, at Detroit, Michigan. The dental department of the Detroit College of Medicine and Surgery has been secured for the sessions. Michigan has generously invited her sister States to share her hospitality and be her guests on this occasion. The programme contemplates four literary sessions, two half-days of clinics and one half-day of hurrah. This latter will come in the form of an excursion thirty-two miles up the Detroit River to the St. Clair Flats, where we will dine at one of the club-houses built on piles in the middle of Lake St. Clair. Special hotel and railroad rates are assured and will be announced later. The railroad fare will be at least as low as one and one-third fares. All reputable dentists in the three States are cordially invited to attend.

EXECUTIVE COMMITTEE.

---

### UNION MEETING.

THE Second Union Meeting of the Washington City Dental Society and the Maryland State Dental Association will be held in Baltimore, April 17 and 18.

A cordial invitation is extended to members of the profession to be present. Special hotel rates will be secured.

W. W. DUNBORACCO,
*Secretary.*

1023 EDMONDSON AVENUE.

